# The Context of Temporal Processing Is Represented in the Multidimensional Relationships between Timing Tasks

**DOI:** 10.1371/journal.pone.0003169

**Published:** 2008-09-09

**Authors:** Hugo Merchant, Wilbert Zarco, Ramon Bartolo, Luis Prado

**Affiliations:** Instituto de Neurobiología, UNAM, Querétaro, México; Harvard Medical School, United States of America

## Abstract

In the present study we determined the performance interrelations of ten different tasks that involved the processing of temporal intervals in the subsecond range, using multidimensional analyses. Twenty human subjects executed the following explicit timing tasks: interval categorization and discrimination (perceptual tasks), and single and multiple interval tapping (production tasks). In addition, the subjects performed a continuous circle-drawing task that has been considered an implicit timing paradigm, since time is an emergent property of the produced spatial trajectory. All tasks could be also classified as single or multiple interval paradigms. Auditory or visual markers were used to define the intervals. Performance variability, a measure that reflects the temporal and non-temporal processes for each task, was used to construct a dissimilarity matrix that quantifies the distances between pairs of tasks. Hierarchical clustering and multidimensional scaling were carried out on the dissimilarity matrix, and the results showed a prominent segregation of explicit and implicit timing tasks, and a clear grouping between single and multiple interval paradigms. In contrast, other variables such as the marker modality were not as crucial to explain the performance between tasks. Thus, using this methodology we revealed a probable functional arrangement of neural systems engaged during different timing behaviors.

## Introduction

The quantification of the passage of time is a ubiquitous and crucial phenomenon in a large repertoire of behaviors. In the hundred of milliseconds range, for example, interval timing is a complex process that is not linked exclusively to a specific sensory modality or motor behavior [1]. It is, however, involved in a broad spectrum of behaviors, ranging from object interception and collision avoidance to musical perception and performance, and it is exhibited by a wide variety of vertebrates including rats, pigeons, and humans [2], [3]. Nevertheless, not all behaviors depend on an explicit timing system where the temporal variability increases as a function of the interval to be timed (i.e. scalar property of interval timing; [4]–[6]). Recent studies have emphasized that in some tasks time is an emergent property of the way in which events are organized during motor activity or within a sensory modality [7], [8]. For example, continuous drawing tasks have been associated with an implicit timing process, since their temporal precision is not correlated with well-known explicit timing tasks, such as multiple tapping and interval discrimination tasks [9]. In addition, the central component of timing variability, measured as the slope from the timing variance plotted against the square of the timed interval, also differed for tapping and drawing tasks [10]. Hence, explicit and implicit timing processes can be clearly dissociated.

Now, psychologists have used different analytical tools, other than psychometric techniques, to study complex perceptual or cognitive processes. For example, without any quantitative information about the physical properties of colors, natural visual scenes, or speech sounds, researchers have learned about how humans process these stimuli using the analysis of ratings of perceived dissimilarity, values by which the stimuli are actually distinguished from each other. These dissimilarities are used in analyses, such as hierarchical clustering and multidimensional scaling (MDS), in order to reveal the most relevant physical dimensions of complex stimuli [11]. In fact, these two methods are designed to study complementary aspects of the underlying psychological structure, starting from pair wise measures of dissimilarity in large groups of complex stimulus comparisons that are summarized in a matrix. On one side, MDS reduces the number of dimensions in large dissimilarity matrices obtaining the most representative multidimensional spatial configuration between data, whereas hierarchical clustering reveals a nondimensional representation in the form of tree structures or dendrograms [12], [13]. Indeed, in the present study we used the same methodology to study the organization of temporal performance in ten different tasks that involved time perception, tapping, or circle drawing, with the purpose of gaining more information about mechanisms governing implicit and explicit timing in a variety of behavioral contexts.

## Results

### General

**Figure 1:**
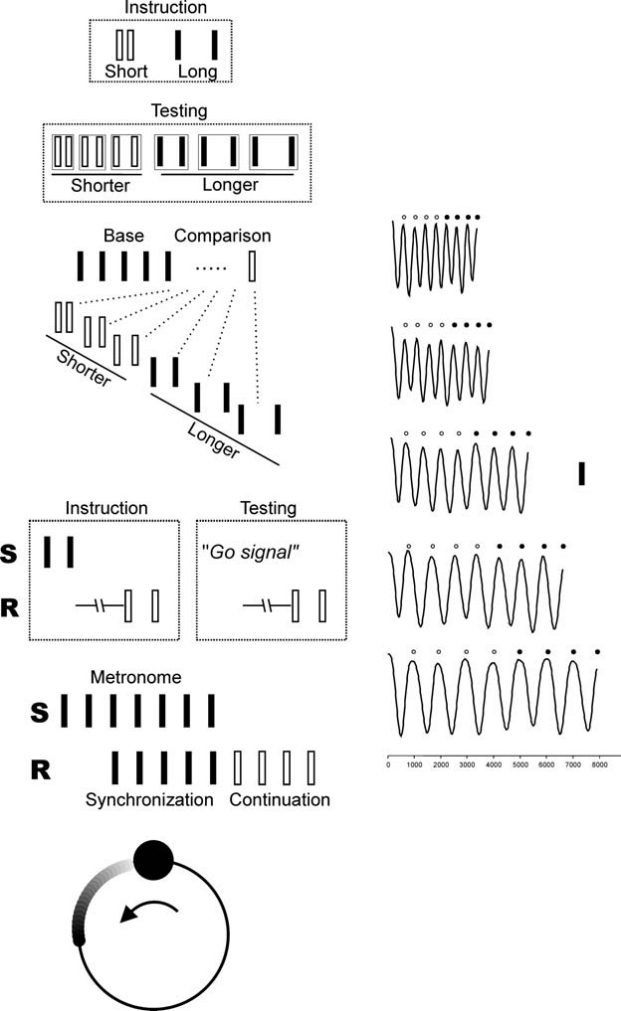
Timing tasks. A. Categorization B. Discrimination C. Single Interval Production D. Multiple Interval Production E. Circle Drawing F. Representative kinematic trajectories for the y-axis in the circle drawing task. The onset of each cycle is marked by the small circles on the top. Open and closed circles correspond to the synchronization and continuation phases, respectively.

The variability of temporal performance of twenty subjects was measured in ten different timing tasks that cover different aspects of behavior. First, these tasks can be grouped in explicit (categorization, discrimination, single interval tapping, and multiple interval tapping) and implicit (circle drawing) timing tasks. In addition, we included three motor (single and multiple interval tapping, and circle drawing), and two perceptual (categorization, discrimination) paradigms that, in fact, can also be subdivided into single (categorization, single interval tapping) or multiple interval (discrimination, multiple interval tapping, and circle drawing) tasks. The tasks included time intervals that were defined by auditory (A) or visual (V) markers (Fig 1). It is very important to emphasize that all the tasks involved temporal processing of the same time intervals (range of 350 to 1000 ms), and that each subject performed all tasks. Therefore, this methodological strategy allowed for a thorough evaluation of temporal and non-temporal components of the subjects' behavior, with a high statistical sensitivity within and between subjects.

An analysis of variance (ANOVA) was performed, using the performance variability as dependent variable and the implicit/explicit, the number of timed intervals, the perception/production, and modality parameters as factors. The results showed, significant main effects for all the factors as follows: implicit/explicit (F_(1,995)_ = 28.79, p<0.0001), the number of timed intervals (F_(1,995)_ = 52.131, p<0.0001), the perception/production (F_(1,995)_ = 169.64, p<0.0001), and modality (F_(1,995)_ = 26.2, p<0.0001). Thus, as depicted in Table 1, the performance variability for the explicit timing conditions was larger in perceptual than in motor-timing tasks, was also larger using visual rather than auditory stimuli, and decreased as a function of the number of intervals. In addition, the temporal accuracy in the circle drawing tasks showed intermediate values between the single and multiple interval tapping tasks. Furthermore, the reliability coefficients for the three production tasks were close to one (Table 1), indicating that the multidimensional analyses below are meaningful with the current data sets.

**Table 1:** Mean and SEM of the performance variability (SD) averaged across subjects and intervals for each task.

Task	Mean	SEM	Reliability
Categorization A	59.84	3.87	
Categorization V	78.47	5.37	
Discrimination A	41.70	2.91	
Discrimination V	70.16	4.66	
Single Interval Tap A	42.02	2.13	0.95
Single Interval Tap V	44.75	2.21	0.96
Multiple Int. Tap A	25.67	0.93	0.98
Multiple Int. Tap V	25.10	1.00	0.97
Circle Drawing A	41.79	1.62	0.97
Circle Drawing V	42.06	1.60	0.97

Reliability coefficients are also shown for the production tasks. A = auditory, V = visual.

It is important to mention that the performance differences between the explicit timing tasks have been reported in detail elsewhere [5]. Here we report the relative relationships in the performance variability between the ten paradigms using multidimensional analyses. Nevertheless, in order to dissociate the performance timing bias from the task multidimensional interrelations, we performed an ANOVA where the constant error ([produced or estimated interval] - target interval) was the dependent variable and the implicit/explicit, the number of timed intervals, the perception/production, and the modality were used as factors. The results showed significant main effects only for the number of timed intervals (single vs multiple) (F_(1,995)_ = 13.7, p<0.0001). The implicit/explicit (F_(1,995)_ = 0.093, p = 0.761), the perception/production (F_(1,995)_ = 2.47, p = 0.116), and the modality (F_(1,995)_ = 0.02, p = 0.888) did not showed significant effects. These properties are evident in Table 2 that shows the constant error mean and SEM for the ten tasks. Additionally, Table 3 shows that the estimated or produced intervals were close to the target intervals in all tasks. Therefore, it is unlikely that the tasks interrelations showed below with multivariate analyzes were due to poor performance in particular intervals or tasks.

**Table 2:** Mean and SEM of the constant error averaged across subjects and intervals for each task.

Task	Mean	SEM
Categorization A	−7.36	4.32
Categorization V	9.05	4.68
Discrimination A	−14.32	3.99
Discrimination V	−11.82	5.06
Single Interval Tap A	5.29	3.43
Single Interval Tap V	−2.09	3.91
Multiple Int. Tap A	3.03	2.08
Multiple Int. Tap V	−13.17	3.15
Circle Drawing A	−11.21	3.84
Circle Drawing V	−4.80	3.70

**Table 3:** Mean (±SEM) of the estimated (PSE, perceptual tasks) or produced (motor tasks) intervals across subjects for each task and interval.

Interval	Cat A	Cat V	Dis A	Dis V	STap A	STap V	MTap A	MTap V	CirD A	CirD V
350	353.3±4.3	360.9±6.3	351.4±4.9	361.3±5.9	353.3±3.3	356.0±4.6	351.3±1.9	341.6±3.3	354.6±3.4	366.4±3.9
450	443.5±5.6	457.3±6.1	447.8±4.7	459.3±6.5	463.5±5.3	456.5±6.8	466.4±2.2	449.0±4.7	468.0±5.1	468.9±3.7
650	639.8±6.6	673.9±11	644.1±7.6	627.3±9.0	653.4±9.4	649.8±9.6	656.2±3.4	641.8±6.9	643.3±6.9	647.2±6.1
850	827.7±11	848.5±10	826.4±10	844.5±12	852.7±8.8	846.2±8.9	851.7±4.9	834.8±7.3	825.2±8.1	830.7±9.8
1000	998.9±15	1004.6±15	958.7±11	948.6±14	1003.6±9.9	980.9±11	989.6±7	966.9±9.7	952.8±9.9	962.8±8.6

A = auditory, V = visual.

### Dissimilarity matrix

**Figure 2:**
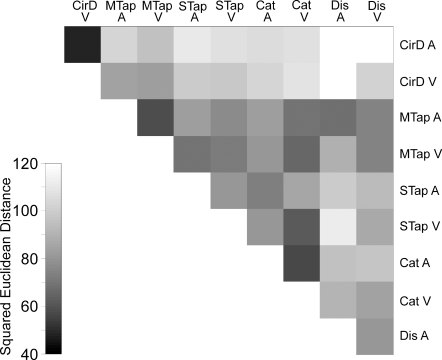
Dissimilarity matrix showing the squared Euclidean distance in a gray-scale (see inset at lower left) for all possible pair-wise task comparisons. The behavioral distance between pairs of tasks follows a simple rule: darker the square, smaller the distance.

Figure 2 shows the dissimilarity matrix of performance variability between the ten tasks. Each square represents the behavioral distance between pairs of tasks with a simple rule: the darker the square, the smaller the distance. In fact, each square corresponds to the squared Euclidean distance between 100-dimensional vectors (20 subjects×5 intervals) associated with the two tasks. It is evident that complex interactions occur between paradigms. However, it is also clear that the circle drawing task, which implies implicit timing, is quite different from the remaining explicit timing tasks. This phenomenon occurred for both sensory modalities (Fig 2).

### Hierarchical clustering dendrograms

**Figure 3:**
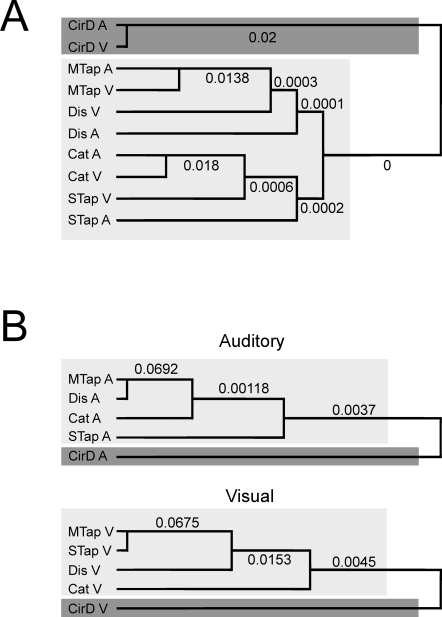
A. Dendrogram for the temporal variability in the ten tasks. The cophenetic correlation coefficient was 0.81. B. Dendrograms for the temporal variability in five tasks where the intervals where marked by auditory (top) or visual (bottom) stimuli. The cophenetic correlation coefficients were 0.87 and 0.76 for the auditory and visual dendrograms, respectively. All the dendrograms show an important segregation between explicit timing tasks (light-gray squares) from the implicit timing paradigms (dark-gray squares). The number on the top of each branch is the probability of the branch occurring by chance.

We used hierarchical clustering with the purpose of classify our tasks in accordance with the distances given in the dissimilarity matrix of Figure 2, following an agglomerative algorithm that starts with each task as a separate cluster or branch. The algorithm, then, merges the closer tasks into successively larger clusters, until only one cluster is left. The resulting clustering pattern is depicted in the dendrogram of Figure 3A, which shows three important features of the behavioral relations between the ten tasks: (1) the circle drawing task, associated with implicit timing, is isolated from all the explicit timing tasks (light gray), having separate branches (dark gray) for both auditory and visual interval markers; (2) the two single interval and the two explicit multiple interval tasks form a bigger branch; (3) the initial clustering was between the same tasks but different modalities, particularly for the circle drawing, categorization, and multiple interval tapping tasks. A bootstrap technique was used to assess the reliability of the tree topology. In fact, the probability that each tree ramification was a random event is shown on top of the branches in Figure 3A. All the branches show significant effects, with a chance likelihood that was less than p = 0.05.

The individual dendrograms for the auditory and visual stimuli, shown in Figure 3B, reveal additional properties of the multidimensional relations between tasks. For example, for auditory markers, the multiple interval tasks (multiple interval tapping, discrimination) are close together forming a branch, whereas the categorization task is the next closest to them, followed by the single interval tapping in another branch. In contrast, the dendrogram for the visual markers clearly shows one explicit timing branch for production (single and multiple interval tapping), followed by the perceptual tasks discrimination and then categorization in other branches. These results suggest that the modality used to define the time intervals has an influence on the level of association between tasks, and that the behavioral relations of the perceptual tasks were the most affected by the modality. Nevertheless, the implicit timing task (circle drawing) again formed a solitary branch separate from the explicit timing tasks in both modality trees.

### MDS analysis

**Figure 4:**
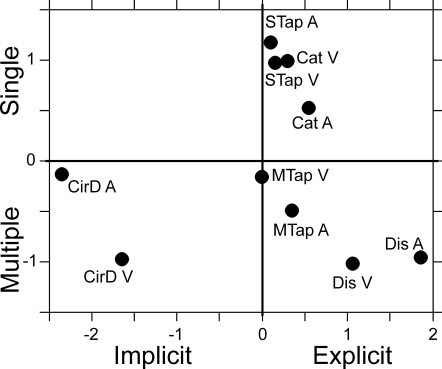
Two-dimensional representation of the temporal performance in the ten tasks, obtained using MDS analysis. The most important dimension (abscissa) separated the circle drawing from all other timing tasks, whereas the second dimension (ordinate) separated single from multiple interval tasks.

The MDS is an analytical method that reduces the dimensionality of a data set, in this case the dissimilarity matrix of Figure 2, to create a two or three dimensional representation of the complex relations between the data. Thus, the goal of the MDS analysis is to detect meaningful underlying dimensions of multidimensional data sets. Our results showed that the MDS analysis was successfully applied to the 9×9 dissimilarity matrix of Figure 2; the stress value was 0.146, and the R^2^ was 0.902 (see Materials and Methods for goodness to fit criteria). The derived configuration plot in 2-D is shown in Fig. 4, where it can be seen that the most important dimension (abscissa) separated the circle drawing from all other timing tasks, whereas the second dimension (ordinate) separated single from multiple interval tasks. Thus, explicit and implicit timing paradigms can be dissociated based on the pair-wise dissimilarities in the temporal performance variability between tasks. In addition, multiple interval tasks, including both implicit and explicit timing behaviors, were distinguished from the categorization and the single interval tapping task. Therefore, engaging a cyclic behavioral loop during multiple interval tasks elicits clear differences in performance from behaviors where only a single interval must be timed.

The bootstrapping technique was also used to generate random dissimilarity matrices from the original data and then carry out MDS analyses. The probability of the original stress solution falling within the distribution of ten thousand random data was less than 0.0087. In addition, the same analysis showed that the implicit-explicit and the single-multiple axes had a probability of being a random event of 0.0172 and 0.0053, respectively.

As a final question, we were interested in finding out whether the implicit-explicit and the single-multiple axes were consistently obtained when a subset of subjects were analyzed using MDS, or whether other superordinate dimensions could be obtained in specific subgroups of subjects. Consequently, we carried out a permutation analysis as follows. The 9×9 dissimilarity matrix based on the temporal performance of twelve of the twenty subjects was computed for all the possible permutations (see Materials and Methods). Then the MDS analysis was carried out as above, and the resulting configuration was saved for each permutation. The results showed that the implicit/explicit axis was found in 88.6% of the twelve subject permutations, whereas in 54.4%, 24.3%, and 0.026% of the permutations the number of timed intervals, the perception/production, and the modality superordinate dimensions were found, respectively. Thus, these results indicate that, in the multidimensional interactions between tasks, the implicit/explicit and single/multiple interval parameters are better represented across all the subjects than the perception/production, and specially the modality components. Indeed, the hierarchical clustering results showed that the marker modality had a relatively small impact in the organization of the task grouping, since it conformed the lowest level of branching. In addition, only the dendrogram for the tasks in the visual modality (Fig 3B, bottom) showed the clustering between perception (categorization and discrimination) and production (single and multiple interval tapping) tasks. Thus, these results stress the complementary nature of the two multidimensional analyses. Both were applied to the dissimilarities in performance variability between tasks. However, the MDS identified the most important behavioral parameters defining the relationships between the tasks, whereas the dendrograms showed a more comprehensive picture of the variables that act as grouping elements between tasks.

## Discussion

This paper illustrates how different types of analytical representations, including multidimensional spatial configurations and nondimensional dendrograms, can reveal important properties of the mechanisms underlying the performance variability in different tasks [14]. At the heart of the approach is the assumption that information contained in the performance variability reflects the proximities or the overlap between distributed neural networks engaged in the prominent behavioral features of each paradigm. Thus, the MDS and cluster analyses are used to reduce the number of dimensions in order to make the configuration of the distributed systems more understandable. In fact, both the hierarchical clustering and MDS analyses validated the distinction between explicit and implicit timing, with a clear separation of the temporal variability of the circle drawing task from the explicit timing tasks (categorization, discrimination, single and multiple interval tapping). Consequently, these results support the notion of different brain processes involved in the execution of behavior over time. On one side there is an explicit representation of the passage of time, and on the other, the temporal properties of the behavior are emergent and depend on mechanisms that may not quantify time in a direct fashion.

The performance dissociation of explicit and implicit timing in repetitive tapping and drawing tasks has been meticulously documented using correlation [7], [9], [15] and slope [10] analyses. For instance, the temporal consistency during a continuous circle drawing task (very similar to our circle drawing) is not correlated with the timing variability during multiple interval tapping, discrimination, or a task where circle drawing is intermittent [7], [9]. It is important to note that the implicit/explicit timing distinction holds independently of the joints employed during drawing, because the subjects in most of the previous studies used the elbow and shoulder [9], [15], whereas in the present study the subjects used the wrist as the main drawing joint. Interestingly, cerebellar lesions severely disrupt the execution of explicit timing tasks, such as multiple interval tapping and intermittent circle drawing, but they do not affect the performance in the continuous circle drawing task [16], [17]. These results not only support the idea that the cerebellum is part of an internal explicit timing system, but also strengthen the hypothesis that continuous rhythmic movements do not engage a timing-specific mechanism. Nevertheless, a precautionary note is in place. Since in the cerebellar patients' study, the spatial accuracy during circular drawing was not reported, it is possible that normal timing in these patients is due to a speed-accuracy trade off, rather that an implicit timing process that emerges from producing a more continuous movement. Therefore, it is conceivable that the behavioral distinction between circle drawing and the other tasks in the present study may be due to factors other than implicit timing, including the prominent spatial component of the motor behavior in this paradigm.

The present results also showed an important segregation in the performance variability between single and multiple interval timing, particularly in the MDS analysis. This suggests that the activation of a cyclic pattern of behavior not only confers an advantage regarding temporal variability and accuracy in multiple interval tasks as reported before [5], [18]–[20], but also may engage a distinctive neural substrate that can be discriminated from the single interval mechanisms using multivariate analytical approaches. Under this scenario, it is possible that the brain mechanisms underlying cyclic behavior, in implicit and explicit timing contexts, have some commonalities that are not shared with one-interval tasks.

On the other hand, the internal consistency analysis using MDS in subsets of subjects, showed that despite the prominence for the representations of the implicit/explicit and number of timed intervals, the perception/production superordinate dimension was also present in the multidimensional relations of temporal variability among more than 25% of the subgroups of twelve subjects. This suggests that the activation of the motor system during a production task elicits differences in performance from perceptual tasks, where timing decisions are expressed by pushing a button. Therefore, it seems reasonable to expect that the ordinate axis in the MDS 2D plot of a subgroup of subjects represents important, but non-temporal, aspects of the variability in the execution of our ten tasks [5].

The marker modality did not create superordinate dimensions in the resulting MDS axes. These results are at odds with studies showing that, in both perceptual and production tasks, visual stimuli produce more variable time estimates than auditory ones [5], [21]–[23], and that the temporal precision increases as a function of the number of intervals to be timed [5], [18]–[20]. However, our present MDS results may reflect the fact that the explicit-implicit and number of timed intervals functional distinctions are more important than the task modality. Indeed, the dendrograms obtained, which showed a more comprehensive picture of the grouping between behavioral parameters, demonstrated some relevance of task modality. For example, in the ten tasks dendrogram the three tasks have individual low-level branches containing both marker modalities, with the exception of the single interval tapping and discrimination tasks that showed two different but close branches for visual and auditory stimuli. In addition, at the level of the individual modality dendrograms we found two important features. The tree associated with auditory markers showed that the multiple interval tasks (discrimination and multiple interval tapping) formed an individual branch, with the categorization closer to them in an individual branch, followed by the single interval tapping in another, more distant one (Fig. 3B). On the other hand, the dendrogram for the visual markers showed one explicit timing branch for production (single and multiple interval tapping), followed by the perceptual tasks discrimination and then categorization in other branches. Hence, these findings suggest that the modality used to define the time intervals has also some influence on the level of association between tasks, and that the functional relations of the perceptual tasks could be affected by the modality, which is a phenomenon that we already described using slope and correlation analyses [5].

One of the current views regarding the neural underpinnings of temporal information processing is that timing depends on a distributed but dedicated clock-like neural mechanism [24]. Indeed, several fMRI studies have described a distributed timing system that includes the cerebellum, as well as the supplementary motor cortex (SMA), dorsal premotor cortex, posterior parietal cortex, putamen, the ventrolateral thalamus, and the dorsal prefrontal cortex [2], [25]–[27]. All these structures are densely connected [28] forming a network. However, these areas have been also associated with other sensorimotor behaviors [29]–[31]. Hence, it is conceivable that the dedicated neural clock may be represented in the dynamic way in which these structures interact and process information [27]. Under this scenario, we can hypothesize that the rules of the network processing may change according to the multivariate relations described in the present paper. Needless to say that elaborate neurophysiological experiments, using a multielectrode and a multiarea approach, are necessary to test this idea.

Overall, the present findings indicate that the functional relationships between timing tasks can be described using the multidimensional dissimilarities of their inherent performance variability.

## Materials and Methods

### Participants

Twenty (10M, 10F) subjects, mean (SD) age of 26.5 (2.5) years, (range: 23–32 years) participated in this study. Additional details about the temporal performance of twelve of the participating subjects in the explicit timing tasks are presented in a preceding paper [5]. They were right-handed, had normal or corrected vision, and were naive about the task and purpose of the experiment. All subjects volunteered and gave written consent for this study before commencement of experiments, which were approved by the National University of Mexico Institution Review Board.

### Apparatus

Subjects were seated comfortably on a chair facing a computer monitor (Dell Optiplex 19”) in a quiet experimental room and tapped on a push-button (4 cm diameter, #7717, Crest, Dassel MN, USA) during the production tasks (see below). In addition, during the perceptual tasks subjects were asked to push a key on the computer keyboard to reflect their decisions. Finally, during the circle drawing task the subjects operated a joystick (H000E-NO-C, CTI electronics, Stratford CT, USA) to control a feedback cursor on the computer screen. The subjects could not see their hand during tapping or circle drawing. The stimulus presentation and collection of the behavioral responses were controlled by a custom-made Visual Basic program (Microsoft Visual Basic 6.0, 1998) on a PC computer. Auditory stimuli were presented through noise-canceling headphones (Sony, MDR-NC50), and the sampling rate of the push-button and the joystick was 200 Hz.

### Task 1: Categorization of time intervals (Cat)

#### a. Experimental task

The subjects were trained first to press the n-key on the keyboard after the presentation of an extremely short interval, or to press the m-key after the presentation an extremely long interval. At least 20 trials (short/long) were performed in this training phase. Categorization feedback was provided during the training phase, with the word ‘correct’ or ‘incorrect’ on the screen. Once the subject learned to associate the short and long intervals with the response on the ‘n’ and ‘m’ key, respectively, intermediate intervals were also presented. Thus, the subject was required to push one of the keys to indicate his/her categorical decision for eight intervals using acquired category boundaries and an implicit middle base interval set during the training period (Fig 1A). The intertrial interval was 1.5 s.

#### b. Stimuli

The stimuli were tones (33 ms, 2000 Hz, 50 dB) or visual stimuli in the form of a green square (4 cm side), presented in the center of a computer screen for 33 ms. The frame-rate of the video board (60 Hz) was accurately calibrated, and the duration of visual presentations was controlled precisely in terms of the number of frames. Eight intervals were used for each of the five different implicit intervals (II [350, 450, 650, 850, and 1000 ms]). For the 350 ms II the intervals were 233, 283, 316, 333, 366, 383, 416, and 466. For 450 ms II the intervals were 299, 366, 416, 433, 466, 483, 533, and 599. For the 650 ms II the intervals were 433, 533, 583, 633, 666, 699, 766, and 866. For 850 ms II the intervals were 566, 666, 783, 816, 883, 916, 1033, and 1133. Finally, for the 1000 ms II the intervals were 699, 816, 933, 966, 1033, 1066, 1183, and 1299. These intervals were carefully chosen to maximize the quality of the threshold boundaries. In all cases, the first four were considered short intervals while the last four were long intervals. Thus, one repetition of the task for each implicit middle base interval included the categorization of the eight intervals. The intervals were presented pseudorandomly for each base interval, and ten repetitions were collected for one implicit middle base interval before moving to the next interval.

#### c. SD calculation

The difference threshold is equivalent to one SD from the implicit standard interval [32], [33]. In order to calculate this threshold a psychometric curve was constructed, plotting the probability of long-interval categorization as a function of the interval. A logistic function was fitted to these data, and the SD was computed as half the difference between the interval at 0.75*p* and that at 0.25*p*. Finally, the point of subjective equality (PSE, 0.5*p*) was considered the estimated interval (see Table 3). Details about the logistic function fitting are given below.

### Task 2: Discrimination of time intervals (Dis)

#### a. Experimental task

The subjects were trained to discriminate between a standard and a comparison interval, pressing the n-key on the keyboard if the comparison interval was shorter, or the m-key if it was longer than the standard interval. On each trial, participants heard a series of six tones (33 ms, 2000 Hz, 50 dB) or viewed six visual stimuli (green squares, 10 cm side, 33 ms). The first five created the four isochronous standard intervals. The sixth one produced the comparison interval that was either shorter or longer than the standard (Fig 1B). Again, 10 trials (extreme short/long) were performed in the training phase, followed by 8 trials for each of the eight standard/comparison combinations. Feedback was provided, with the word ‘correct’ or ‘incorrect’ on the screen during the training phase. The intertrial interval was 1.5 s.

#### b. Stimuli

The intervals used in the categorization task were also used in this task as comparison for each of the five different standard intervals (350, 450, 650, 850, and 1000 ms). One repetition of the task for each standard interval included the discrimination of the eight intervals, and 8 repetitions were collected. In addition, in 20% of the trials the standard and comparison intervals were chosen at random within the range of 330 ms to 1100 ms. This was done with the purpose of maintaining the subject's attention to both interval durations across all trials. Finally, the comparison intervals were presented pseudorandomly within each standard interval, and the order between standard intervals was chosen randomly.

#### c. SD calculation

The SD was calculated in the same fashion as in the categorization task.

### Task 3: Production of a single time interval (STap)

#### a. Experimental task

For each interval there was a training and an execution period (Fig 1C). In the training period, a target interval (two stimuli separated by an interval of a particular duration) was presented at the beginning of the trial. Then the subject tapped twice on the push-button to produce the same interval. This was repeated for 5 training trials, after which the subject entered the execution period, where he/she produced another 10, single intervals after a go signal appeared on the screen. Again, feedback was displayed on the screen, indicating the subject's intertap interval and SD across trials of the same interval. The intertrial interval was 1.5 s.

#### b. Stimuli

The stimuli were tones (33 ms, 2000 Hz, 50 dB) or visual stimuli in the form of a green square (4 cm side) presented in the center of a computer screen for 33 ms. The interval durations were 350, 450, 550, 650, 850, or 1000 ms. Ten trials during the execution period were collected for a particular interval duration before changing to another one. The intervals were chosen pseudorandomly.

### Task 4: Production of multiple time intervals (MTap)

#### a. Experimental task

Subjects produced tapping movements on a push-button device synchronized to a sensory stimulus and then were asked to continue tapping with the same interval without sensory stimulus (Fig 1D). At the beginning of the trial, the stimuli were presented with a constant interval. Subjects were required to push a button each time a stimulus was presented, which resulted in a stimulus-movement synchronization. After five consecutive synchronized movements the stimulus was eliminated, and the subjects continued tapping with the same interval for four additional intervals. Feedback was displayed on the screen, indicating the human subject's mean intertap interval and the SD for the continuation phase of the trial. The interval separating the synchronization and the continuation phase was not included in this feedback measure or in the further analyses. The intertrial interval was 1.5 s.

#### b. Stimuli

The same stimuli and interval durations as for the single interval tapping were used. The intervals were chosen pseudorandomly, and ten repetitions were collected for each interval.

### Task 5: Circle Drawing (CirD)

#### a. Experimental task

The subjects operated a joystick, a vertical rod placed in front of the subject at midsagittal level that controlled a feedback cursor, which was displayed in the monitor as a circle of 0.55 cm in diameter. At the beginning of the trial, the subjects had to place the cursor within a white circle of 1 cm diameter (“start window”). Then, the stimuli were presented with a constant interval, and the subjects were required to draw a circle with the cursor, following a circular path of 5 cm diameter, during that interval (Fig 1E). Thus, the subjects attempted to pass the feedback cursor through the start window coincident with the presentation of the synchronous stimuli, while continuously moving around the circumference of the path circle. After the drawing of four synchronized circles the stimuli stopped, and the subjects continued to move as consistently as possible at the rate of the extinguished stimuli for another four loops (Fig 1F). Feedback was displayed on the screen, indicating the human subject's mean interloop interval and SD for the continuation phase of the trial. Temporal accuracy was stressed over the spatial accuracy of drawing. Subjects performed the drawing mainly with the wrist joint.

#### b. Stimuli

The same stimuli and interval durations as for multiple interval tapping were used. The intervals were chosen pseudorandomly and eight repetitions for each interval were collected.

#### c. SD calculation

We defined the start of a circle drawing as the point of maximum displacement in the y-dimension (Fig 1F; [9]). We used an algorithm written in Matlab (MathWorks, Natick, MA, Version 7.3.0.267) to determine all local maxima in the y dimension during the synchronization and continuation epochs of the circle drawing task. With these values we determined the mean and SD of the intervals produced during the continuation phase. Again, the interval separating the synchronization and the continuation phase was not included in the analyses.

### Timing Task Procedure

The first twelve subjects performed tasks 1 to 4 in random order in four sessions, followed by the circle drawing in a fifth session. The remaining eight subjects performed the five tasks in random order in the five sessions. At least eight repetitions were collected for each condition and task. Before data collection, practice trials were given in the five tasks until the subjects acknowledged that they understood the tasks and were comfortable with their performance.

### Analysis

#### Logistic regression

This regression was used for the psychometric data of tasks 1 and 2, and is given by:
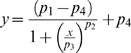
(1)where *p_1_* and *p_4_* correspond to the maximum and minimum values of *y*, *y* is the probability of long interval categorization, *p_2_* is the estimated slope, and *p_3_* corresponds to the value of *x* (time interval) at half of the maximum value of *y*. The percentage of variance explained (*R^2^*) was greater than 90% in all the fittings.

#### Reliability analysis

We computed reliability values (varying from zero to one) for the produced intervals in the single and multiple interval tapping, and circle drawing tasks, using the correlation coefficient from odd and even trials of the same subject and interval. The SPSS statistical package (version 12, SPSS Inc., Chicago, IL 2003) was used for this purpose.

#### Hierarchical cluster analysis

We performed hierarchical clustering analyses [12] to determine the pattern of grouping of the temporal variability associated with five tasks where the intervals were defined by auditory or visual markers. The SD of temporal performance, for each time interval and task, was standardized and re-expressed as a z-score within each task. Thus, the primary clustering variables consisted of 100-dimensional vectors for each task, containing the z-scores for each subject (n = 20) and interval (n = 5). The squared Euclidean distance between the 100-dimensional vectors of all possible pairs of tasks formed a 9×9 dissimilarity matrix (Fig. 2) that was used for both the hierarchical cluster and MDS analyses. Dendrograms were obtained as a result of the agglomerative algorithm in the clustering analysis. The cophenetic correlation coefficient was computed to establish the goodness of fit of the clustering (Matlab, MathWorks, Natick, MA, Version 7.3.0.267). In fact, cophenetic correlation coefficient is defined as the linear correlation coefficient between the distances obtained from the tree and the original distances (or dissimilarities) used to construct the tree.

In order to determine the significance of each of the tree branches (Fig. 3) a bootstrapping technique was performed as follows. First, for each subject and time interval, the value of the temporal SD was permuted among the 10 tasks. Second, the 100×10 matrix was re-expressed as z-scores within each task, and then a hierarchical clustering analysis was performed on the corresponding dissimilarity matrix. The resulting tree configuration was saved. This procedure was repeated 10,000 times, and the number (and percentage) of branches that showed the same original clustering was computed. This analysis was carried out on Matlab (MathWorks, Natick, MA, Version 7.3.0.267) with subroutines for bootstrapping phylogenetic trees (Bioinformatics Toolbox).

#### MDS analysis

The MDS was also performed on the 9×9 dissimilarity matrix, with an ordinal scale (i.e. non-metric MDS; [12]) and two final dimensions (ALSCAL procedure). The success of the MDS analysis was evaluated by computing Kruskal's stress formula 1 and the R^2^. The latter is the proportion of variance of the scaled data (disparities), which is accounted for by their corresponding distances. The SPSS statistical package (version 12, SPSS Inc., Chicago, IL 2003) was used for all the statistical analyses. It is important to note that two assumptions are made with the MDS model: (1) that the appropriate metric for the similarity space between timing tasks is Euclidean and (2) that each set of individual subject data included in the analysis can be modeled by linear stretching of the centroid configuration, as specified by the individual subject weights. If these assumptions hold true, one expects low stress values for the overall MDS solution. In fact, Monte Carlo studies suggest that stress values below 0.2 are indicative of an output configuration with a good fit to the similarity data [34].

An additional set of analyses was carried out with the purpose of determine the representation of different MDS superordinate dimensions or axes throughout subpopulations of the studied subjects, as follows. First, the 9×9 dissimilarity matrix for twelve of the twenty subjects was computed for all the possible permutations:
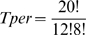
where *Tper* is the total number of permutations and is equal to 125970. Second, the MDS analysis was performed for each dissimilarity matrix. Finally, the resulting MDS configuration was saved for each permutation, and the following criteria were used to define a particular axis. First, the length of an axis was defined as the distance between the maximum and minimum coordinate values for the ten tasks. Then, a superordinate dimension was defined when the distance between the groups of tasks forming an axis (implicit/explicit, single/multiple, perception/production, or auditory/visual) was larger than 1/5 of the total length of that axis. For example, taking the data of Figure 4, the implicit-explicit superordinate dimension was defined when the distance between each CirD task and the other eight tasks was larger than 0.86 (4.3/5), whether for the x or the y axes.
